# Venous Thromboembolism Rate in Patients With Bladder Cancer According to the Type of Treatment: A Systematic Review

**DOI:** 10.7759/cureus.22945

**Published:** 2022-03-08

**Authors:** Omar Abdullah, Deepak Parashar, Israa J Mustafa, Annie M Young

**Affiliations:** 1 Warwick Medical School, University of Warwick, Coventry, GBR; 2 Mosul Cancer Control Centre, Nineveh Health Directorate, Mosul, IRQ

**Keywords:** chemotherapy, cystectomy, bladder cancer, cancer, thrombosis, venous thromboembolism

## Abstract

Bladder cancer (BC) is classified as a high-risk tumour type for venous thromboembolism (VTE). VTE presents an extra challenge in the management of patients with cancer, given the increase in morbidity and mortality on having both conditions.

To summarise the contemporary evidence on the VTE rate in patients with BC according to the stage, type of anti-cancer treatment and highlight VTE rate in the UK and other countries. A systematic review was carried out, and an electronic search for publications between January 2000 and November 2021 was done. Studies recording VTE in BC patients were included, whilst paediatric patients, case reports, studies reporting on a mix of arterial and venous thrombosis, studies reporting DVT or PE only and recorded hospitalised VTE only were excluded. The rate of VTE, country of origin, risk factors and thromboprophylaxis duration for VTE in BC patients were identified.

A total of 38 papers met the search criteria. All publications were original research papers (cohort studies). The overall VTE rate in patients with BC was estimated at 1.9% to 4.7%. For those patients undergoing cystectomy, the VTE rate ranged from 3% to 17.6%; however, the VTE rate in the metastatic stage of BC patients ranged from 3.1% to 5.1%.

The rates of VTE in BC patients are high, further increased by interventions such as surgery and chemotherapy. Thromboprophylaxis measures should be optimised. This review highlighted the fact that the VTE rate in BC varies between studies due to the heterogeneity of risk factors reported.

## Introduction and background

Venous thromboembolism (VTE) is a disorder in which a thrombus forms, mostly in the deep veins of the lower or upper limbs, and is called deep vein thrombosis (DVT). The thrombus may travel to the pulmonary arteries where it lodges, causing a pulmonary embolism (PE). Together, DVT and PE are known as VTE. Cancer is an independent risk factor for VTE [1], and VTE is a major cause of morbidity and mortality in patients with cancer [2]. VTE negatively affects the quality of life and increases the risk of further complications such as recurrent VTE and bleeding [3]. In addition, the utilisation of health care resources in cancer patients who have VTE is high [3]. Cancer patients have a five to six-fold increased risk of developing VTE compared with the general population; this can reach seven-fold for cancers of the brain, ovary and pancreas [4]. The pathogenesis of VTE in cancer patients is complex, related to the interaction between the procoagulant properties of the malignant cells themselves, the haemostatic system and characteristics of the patient [5].

BC is categorised as a high-risk tumour type (for VTE) [6]. These authors found the six-month cumulative incidence of VTE in metastatic urothelial BC is 8.2%. VTE rates in patients with BC who have had a cystectomy range between 0.3% and 17.6% [7-8]. Furthermore, in a USA study, exploring VTE in BC patients, the VTE rate was 7.5% (5.5-9.9%) in USA and 3.8% (2.5-5.4%) in non-USA countries, P = 0.005. The standard treatment for muscle-invasive non-metastatic bladder cancer is cystectomy or multimodality treatment (surgery - transurethral resection of bladder tumour plus chemo-radiotherapy). Muscle-invasive BC is proven to increase the risk of VTE, and this risk becomes even higher after cystectomy [9].

This systematic review was undertaken to summarise the contemporary evidence on the VTE rate in patients with BC according to the type of anti-cancer treatment and highlight the VTE rate in the UK vs other countries.

The risk of VTE in BC may vary between different countries due to different guidelines followed in BC management, e.g. European Society for Medical Oncology (ESMO) guidelines are generally used in Europe, National Comprehensive Cancer Network (NCCN) guidelines are used internationally, in particular in the USA and National Institute for Health and Care Excellence (NICE) guidelines are used in the UK, for example, using neoadjuvant chemotherapy or not before cystectomy. Different treatment regimens are preferred in different countries, e.g., the USA and using thromboprophylaxis for 14 or 28 days post cystectomy [10].

BC patients who are undergoing major surgery, e.g., radical cystectomy, are particularly at high risk for VTE; thus, most guidelines recommend pharmacological thromboprophylaxis for patients starting before surgery and continuing for four weeks post-surgery. Thromboprophylaxis for ambulatory BC patients who are receiving chemotherapy is not widely adopted or recommended by guidelines unless the patients have other risk factors [10].

It is important to have evidence on VTE incidence in patients with BC according to known confounding variables for VTE, in the UK and other countries, so that healthcare professionals and patients are informed and can take appropriate action. In practice, optimizing treatment plans to decrease VTE risk while also reducing bleeding risk is significant to patients and the healthcare system; thus, the decision to use pharmacological thromboprophylaxis needs a balance between the risk of VTE and major bleeding [11].

The clinician needs to consider the balance between the risk of VTE and the risk of bleeding before prescribing pharmacological thromboprophylaxis [9]. This systematic review also reveals the rate of VTE in BC patients according to the treatments and length of thromboprophylaxis in the UK and other countries to inform the optimisation of thromboprophylaxis

Aims and objectives of systematic review

Aims

The aims of this review are to find (a) the incidence rate of venous thromboembolism (VTE) according to the anti-cancer treatments received, in and out of the UK; (b) the VTE rate differences between the UK and global data.

Objectives

To explore (a) the VTE rate in BC patients in the UK and other countries; (b) the evidence available on VTE rate in non-muscle-invasive, muscle-invasive, and metastatic BC patients in the UK and non-UK countries; (c) the influence of the duration of pharmacological thromboprophylaxis on VTE rate in patients with BC undergoing cystectomy; (d) the knowledge gaps of VTE in BC that demand further studies in the UK and non-UK countries.

Methods for systematic review

The Centre for Reviews and Dissemination (CRD) Guidance for Undertaking Reviews in Healthcare (CRD, 2008) was utilised to guide this systematic review. A search was conducted on the PubMed and Embase electronic databases (Appendix 1). In addition, conference proceedings of the American Society of Clinical Oncology and the American Urological Association, the two largest international meetings of cancer and urology, were screened for potentially relevant records.

Search inclusion and exclusion criteria

The main outcome of interest was any VTE, which included symptomatic or incidentally detected DVT and PE, diagnosed in patients with BC.

Inclusion criteria included (a) Primary research that confirmed the diagnosis of DVT and/or PE in BC patients; (b) Papers in the English language; (c) Papers published from January 2000 until November 2021.

Exclusion criteria included: (a) Case reports; (b) Studies reporting DVT or PE only; (c) Studies that recorded in-hospital VTE only; (d) Studies occasionally reporting on VTE as one of the adverse effects of surgery or chemotherapy; (e) Studies reporting on a mix of arterial and venous thrombosis as a composite endpoint or lack of clarity on venous thrombosis only.

Data sources and searches

The Preferred Reporting Items for Systematic Reviews and Meta-analysis (PRISMA) guidelines and checklist were followed to develop this review [12]. The following terms were used: ((‘bladder cancer’ OR bladder carcinoma’ OR ‘bladder tumour’ OR ‘bladder urothelial carcinoma’ OR ‘transitional cell carcinoma’) AND (‘VTE’ OR ‘DVT’ OR ‘PE’ OR ‘venous thromboembolism’ OR ‘thrombosis’ OR ‘thromboembolism’ OR ‘deep vein thrombosis’ OR ‘pulmonary embolism’ OR ‘lung embolism’)).

Publications from January 2000 to June 2020 were included (Appendix 1). Data on overall VTE incidence in BC patients were extracted from all related publications. These were the source data for the primary objective - incidence of VTE in BC patients. Reference list checking was carried out to identify further relevant studies. All titles and abstracts from the search were evaluated independently by two reviewers (DP and IM) and disagreements were resolved by a third arbitrator (AY).

Study quality assessment for systematic review

For each of the included studies, the risk of bias was evaluated using the Newcastle-Ottawa Scale (NOS) and Agency for Healthcare Research and Quality (AHRQ) standards and classified as good, fair, and poor. The NOS assigns up to a maximum of nine points for the least risk of bias in three domains: 1) selection of study groups (four points); 2) comparability of groups (two points); and 3) ascertainment of exposure and outcomes (three points) for cohort studies.

A maximum score of 2 can be awarded for comparability and a maximum score of 1 can be given for each of the remaining points. A study can have the maximum possible quality score of 9. Thresholds for converting the NOS to AHRQ standards were applied to have a clear view of quality assessment for each study (Appendix 2).

Ethical considerations for systematic review

Ethics committee approval was not required for this systematic review, as this methodology utilises only previously published material, is not primary research and does not involve humans or data collection.

## Review

Results of the systematic review

Descriptive Characteristics

**Figure 1:**
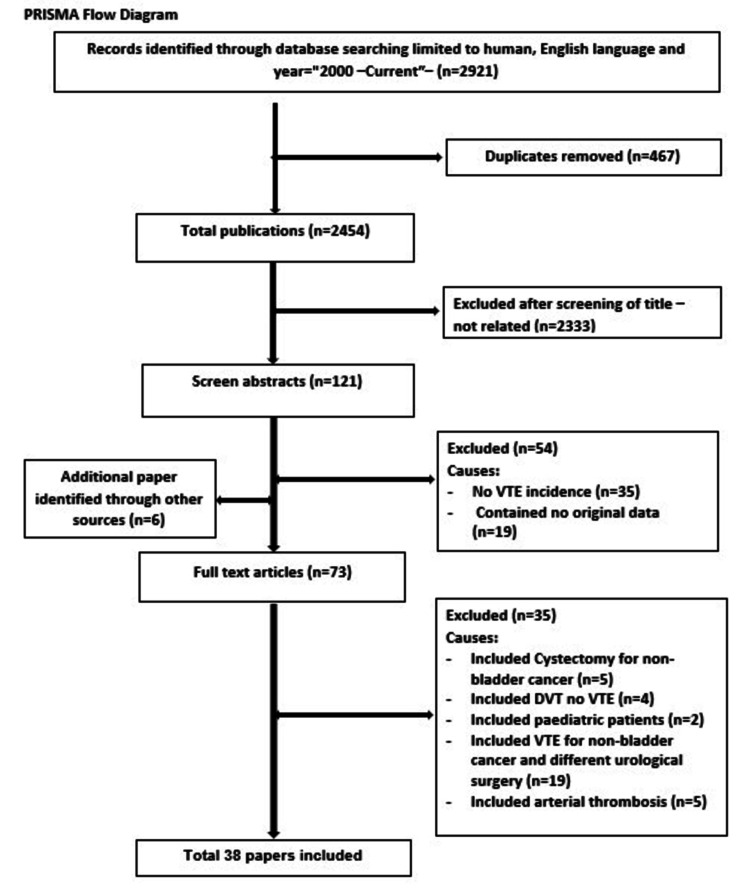
Study selection process of systematic review

Of the 2921 publications, a total of 38 studies met the inclusion criteria and were selected for this systematic review. All stages of the extraction process are shown in the PRISMA flow diagram (Figure 1).

**Figure 2:**
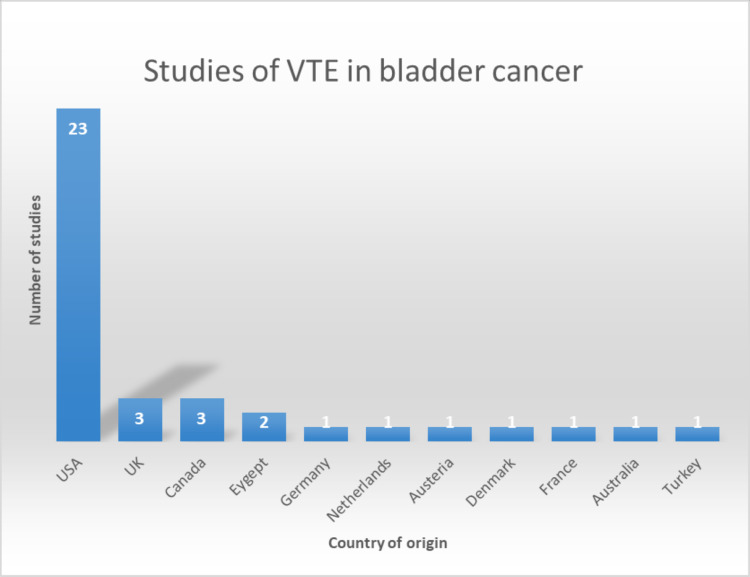
Summary of studies according to country of origin

In the current systematic review, all papers with one exception have good and fair quality according to the Newcastle-Ottawa Scale and AHRQ standards. All publications were original research papers (cohort studies) in which the VTE rate was clearly stated. The cohort studies were generated in different countries: ‘USA’, ‘Canada’, ‘Germany’, ‘France’, ‘Denmark’, ‘Netherlands’, ‘UK’, ‘Australia’, ‘Turkey’, and ‘Egypt’. The majority of the studies were carried out in the USA. The country of origin is summarised in Figure 2.

**Figure 3:**
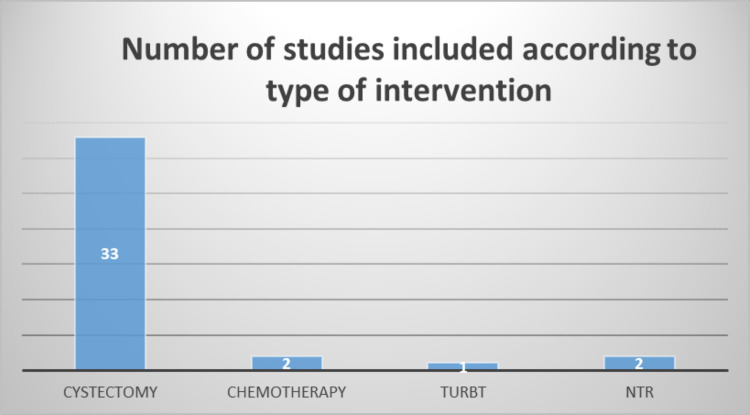
Summary of VTE and BC studies by treatment VTE: venous thromboembolism; BC: bladder cancer

The majority of papers included patients who had undergone cystectomy (n=33) (Figure 3). The further risk of VTE in patients receiving neoadjuvant or adjuvant chemotherapy (with curative intent) was only mentioned in four of the 33 papers [13-16]. There are no data on VTE in patients having multimodality treatments. Only two papers discussed the VTE risk in patients receiving chemotherapy only [17-18]; two papers compared VTE rates in using thromboprophylaxis (TP) for two weeks and an extended duration of four weeks [19-20]; one paper included patients having a transurethral resection bladder tumour (TURBT) only [21].

VTE rate in patients with bladder cancer

This review identified 38 studies specifying the VTE rate in BC patients, with a total of 172,380 BC patients being included, according to the type of treatments (Figure 3) and country of origin; however, two studies stated the overall VTE rate with no data on anti-cancer therapies or TP regimes [22-23]. A cohort study conducted in the UK explored the VTE rate in all cancer patients, including 3152 BC patients, and the absolute rate per 1000 person-years of VTE in BC was 15 [24]. In the US, Sandhu et al. (2010) carried out a retrospective cohort study for 24,861 patients with BC to look at the VTE rate over six years. The one-year and two-year rate of VTE after cancer diagnosis was 1.6% and 1.9%, respectively [22]. Taking both studies together, VTE rates in BC patients were an average of 1.9% to 4.7% (mean = 3.3%) within two years of BC diagnosis. The VTE rate in BC patients in the UK was more than double in the USA.

VTE rate in non-muscle-invasive bladder cancer undergoing transurethral resection bladder tumour: The effect of minimally invasive surgery, TURBT, on the VTE rate in BC patients has rarely been investigated. A single study used the Surveillance, Epidemiology and End Results (SEER)-Medicare linked database, which explored the VTE rate in patients who had TURBT for non-muscle invasive bladder cancer patients. The study reviewed data from 1988 to 2009 for 50,125 patients treated with TURBT; 2.8% of patients experienced VTE within 90 days post-TURBT [21]. In non-muscle-invasive BC undergoing TURBT, no thromboprophylaxis was utilised due to a lack of evidence on the VTE risk as well as concerns around bleeding.

VTE in patients with bladder cancer undergoing cystectomy: The data from 33 studies of patients with BC undergoing cystectomy (invasive surgery) were extracted to find the rate of VTE in patients undergoing cystectomy alone and patients having a cystectomy plus chemotherapy and in and out with the UK. The VTE rates at different time points post-cystectomy were identified: 30 days, 90 days, 180 days, one year or two years. All studies included found an elevated risk of VTE among patients undergoing cystectomy. The rates of VTE in BC patients who had cystectomy were between 1.3% and 23.2% (mean=4.9%). In the UK, two studies explored the rate of VTE in patients undergoing cystectomy and the VTE rates were 2.5% and 2.9% mean=2.7% [25-26]. Three Canadian studies and one from Turkey demonstrated a VTE rate of BC patients treated with chemotherapy and cystectomy of between 1.3% and 9% (mean=5.1%). There were no studies that explored the VTE rate in BC patients having cystectomy and chemotherapy.

Regarding thromboprophylaxis post-cystectomy, 30 studies did not clearly discuss thromboprophylaxis regimens for BC patients undergoing cystectomy. However, two studies from the USA explored thromboprophylaxis effectiveness in BC patients with cystectomy in the post-surgical period. These two studies grouped patients by receiving thromboprophylaxis for 14 days or 28 days post-cystectomy. The patients were followed for 90 days duration. A substantial reduction in VTE rate (from 17.6% to 5%) was recorded in the extended thromboprophylaxis (28 days) group in comparison to the control group (14 days); extended chemoprophylaxis significantly reduced the incidence of VTE (P-value=0.021) [20]. No UK studies explored the impact of using extended thromboprophylaxis on the VTE rate in BC patients who have had cystectomy.

VTE in bladder cancer patients undergoing chemotherapy: Only two studies exploring the VTE in muscle-invasive BC who received chemotherapy were found, and the VTE rates were 5.1% and 8.1% (mean=6.6%) [17-18]. The VTE rate within six months from diagnosis of metastatic urothelial cancer (not specifically bladder cancer) is 3.2%, and the VTE rate in metastatic urothelial cancer and receiving chemotherapy is 5.1% [18]. No data exclusively relating to patients with BC who have had chemotherapy only were found to assess the benefits or drawbacks of thromboprophylaxis in this population.

**Figure 4:**
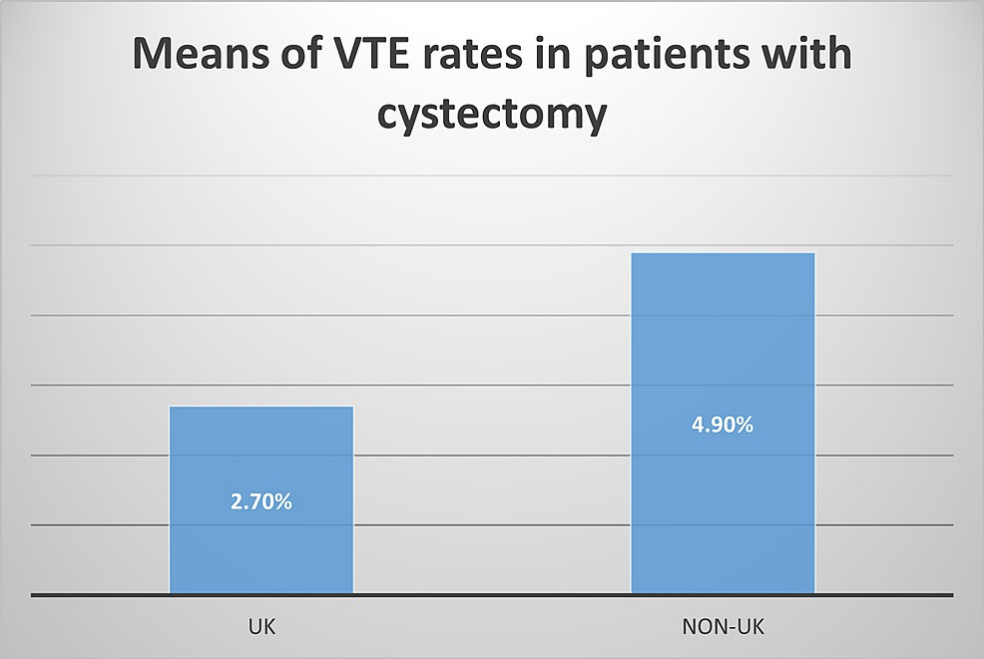
Mean of VTE rates of BC patients who have had cystectomy in the UK and other countries VTE: venous thromboembolism

The mean of VTE rates in BC patients who had undergone radical cystectomy in the UK was lower than in non-UK countries as a group (Figure 4).

Discussion of the systematic review

This review explored the mean of VTE rates within and outside the UK and found the difference with the mean of VTE rates in non-UK countries according to the types of treatments or VTE risk factors in patients with BC. The VTE rates were explored in BC patients according to the risk factors impacting the rate of VTE such as surgery, chemotherapy and thromboprophylaxis in the UK and non-UK countries, where possible.

The review of the VTE rate in patients with BC elicited 38 prospective and retrospective studies that demonstrated VTE rates in different follow-up periods among BC patients who have had a radical cystectomy. Each study has a different rate of VTE and therefore a wide range of VTE rates in patients having cystectomy is noted (1.3% to 23.2%) in this current review. Regarding UK studies, they were only two studies that collected data on the VTE rate in BC patients who have had cystectomy in the UK, and these studies were carried out some years ago (2012 and 2013). Hence, due to the lack of new and enough studies (more than four studies) in recent years, it would be unlikely to be beneficial to carry out a meta-analysis of VTE rate in BC patients based on country (the UK vs non-UK countries). It is important to undertake new studies exploring the VTE rate in BC patients in the UK.

A previous systematic review was carried out on the risk of VTE and bleeding in patients having open radical cystectomy [9]. Many studies included in Tikkinen’s systematic review were occasionally reported the VTE rate as one of the adverse effects of surgery, and it seems that these studies did not check for VTE events carefully. These data may be at risk of information bias [9].

There are many possible explanations for the large variations in the rates of VTE observed within this group: for example, the heterogeneity of risk factors in this population that could affect the rate of VTE such as minimal invasive (robotic) cystectomy or open radical cystectomy [10]. Additionally, 33 of the included studies did not state, discuss or categorise BC patients according to confounding factors that alter the rate of VTE such as receiving surgical thromboprophylaxis for 14 or 28 days, which then makes it difficult to compare rates between studies [13,15-16,25-39,40-53]. All included studies, with the exception of two [19-20], did not clearly discuss or state the thromboprophylaxis measures employed. Having different thromboprophylaxis protocols may be the cause of variation in VTE rate between studies and countries using extended thromboprophylaxis 28 days post-surgery decrease the VTE rate in BC patients with cystectomy.

The timing of follow-up differs between surgical studies. The minimum follow-up duration of patients undergoing cystectomy was 28 days, and the VTE rates were 3% -7.7% (mean=5.6%). In this review, we excluded studies that only drew on data relating to VTE during in-hospital admission because the vast majority of VTEs (82.6%) occur after discharge from the hospital; in other words, there was an inadequate follow-up for VTE in patients undergoing cystectomy [28]. In our current review, the data from three studies that followed up the patients for three months after cystectomy revealed that VTE rates were still high; this may be due to patients having further treatment and hospitalisation [15,18,50]. According to the study by Wallis et al. (2017), the VTE rate peaks at 20 days after cystectomy in BC patients; however, patients continue to be at risk of VTE long after surgery [16]. As found in this review, BC patients who had undergone cystectomy have an elevated risk of VTE for up to three months after surgery [13]. Consideration should thus be given to offering thromboprophylaxis measures and caring for longer than 28 days post cystectomy, in particular, if chemotherapy has been added to the treatment plan or patients have other risk factors for VTE. The majority of BC guidelines typically suggest extended thromboprophylaxis for pelvic surgery, including cystectomy for up to four weeks post‐discharge [51]. Interestingly, the mean of VTE rates in patients who have had cystectomy with chemotherapy is 5.0% [13,15,34], suggesting that systemic chemotherapy did not increase the VTE rate more than the already increased rate imposed by surgery.

Brennan and his team (2018) also mentioned that chemotherapy is not associated with an increased risk of VTE after RC [15]. The presence of metastatic disease increases the VTE rate to the same extent as cystectomy by 5.1% [15]. However, patients with metastatic BC who did not receive chemotherapy had an absolute VTE incidence rate of 3.2% [18]. The VTE rate in patients with metastatic BC who receive systemic chemotherapy further increased to 5.1% within six months. VTE rates based on the chemotherapy group demonstrated no statistical difference when gemcitabine/cisplatin was used as the comparator [18].

Gopalakrishna et al. (2016) mentioned that the VTE rate differed significantly by country of origin among BC patients [53]. This is likely to be due to differences in patients’ characteristics, using preoperative chemotherapy, recording issues and using different protocols of thromboprophylaxis. Thus, it is of value to find the VTE rate in the UK and not depend on the data from non-UK countries, in order to scope the problem and strive to protect the patients from this debilitating condition. The VTE rates were 2.5% and 2.9% within one-year post-cystectomy as the UK study found [25-26].

Table 1 lists the studies that provided information on the VTE rate in bladder cancer patients receiving thromboprophylaxis while Table 2 lists those included information on the VTE rate in bladder patients undergoing cystectomy and/or chemotherapy treatment.

**Table 1:** VTE rate in bladder cancer patients receiving thromboprophylaxis TP: thromboprophylaxis; ETP: extended thromboprophylaxis; Cyst.: cystectomy; VTE: venous thromboembolism

References	Study origin	VTE rate (%) with ETP	VTE rate (%) without ETP	Follow-up (days)	Enrolment period	Participants	Type of study	Interventions	Quality
Schomburg et al. [20]	USA	5	17.6	90	2012-2015	79-51	Retrospective	Cyst. ± TP	Good
Pariser et al. [19]	USA	5	12	90	2011- 2014	402-234	Retrospective	Cyst. ± TP	Good

**Table 2:** Studies included information on VTE rate in bladder patients undergoing cystectomy and/or chemotherapy treatment CT: chemotherapy; Cyst.: cystectomy, NCT: neoadjuvant chemotherapy; INT: international; TURBT: transurethral resection bladder tumour; ACT: adjuvant chemotherapy: NA: not applicable; VTE: venous thromboembolism

References	Study origin	VTE rate (%)	Follow-up (days)	Enrolment period	Participants	Study type	Intervention	Quality
De Martino et al. [27]	USA	4.9	30	2007- 2009	307	Retrospective	Cyst.	Fair
Alberts et al. [28]	USA	5.5	30	2005- 2012	2065	Retrospective	Cyst.	Fair
Soave et al. [29]	Germany	3	30	2007- 2014	201	Retrospective	Cyst.	Poor
Vukina et al. [30]	USA	5.7	30	2005- 2011	878	Prospective	Cyst.	Fair
Mossanen et al. [31]	USA	5.5	30	2007- 2012	8671	Retrospective	Cyst.	Fair
Chen Emily et al. [32]	Australia	7.7	30	2009- 2013	53	Retrospective	Cyst.	Fair
Lyon et al. [33]	USA	4.2	30	2011- 2016	8241	Retrospective	Cyst.	Fair
Van Dlac & Cowan [34]	USA	6.0	30	2005- 2011	1307	Retrospective	Cyst.	Good
Zaffuto et al. [21]	USA	2.8	90	1988- 2009	50125	Retrospective	TURBT	Good
Daneshmand et al. [35]	USA	5.4	30	2011-2012	110	Prospective	Cyst.	Good
Cookson et al. [36]	USA	1.3	30	1995-2000	304	Retrospective	Cyst.	Poor
Breau et al. [37]	USA	3.2	30	2006-2012	2303	Retrospective	Cyst.	Fair
Tyson et al. [38]	USA	6.8	30	2005-2011	1792	Retrospective	Cyst.	Good
De Vries et al. [39]	Netherlands	2.3	30	2007-2008	85	Prospective	Cyst.	Good
Nguyen et al. [14]	Turkey	1.3	30	2011-2015	74	Prospective	Cyst. + CT	Poor
Brossner et al. [40]	Austria	3.2	30	1998-2002	92	Retrospective	Cyst.	Fair
Hugen et al. [41]	USA	4.4	90	1985 - 2015	2694	Retrospective	Cyst.	Fair
Dorion et al. [13]	Canada	5.4	90	1994 - 2008	3879	Retrospective	Cyst. + CT	Good
Sun et al. [42]	USA	4.7	90	1971-2012	2316	Retrospective	Cyst.	Good
Khan et al. [26]	UK	2.5	90	2003-2008	158	Retrospective	Cyst.	Good
Fairey et al. [43]	Canada	3.5	90	2000-2006	314	Retrospective	Cyst.	Good
James et al. [44]	USA	6.3	90	2008-2010	2565	Retrospective	Cyst.	Good
Laymon et al. [45]	Egypt	4	90	2004-2014	1737	Retrospective	Cyst.	Good
Brennan et al. [15]	Canada	9	180	1994-2013	4205	Retrospective	Cyst. + CT	Good
Ramos et al. [18]	USA	5.1	180	2000-2013	1762	Retrospective Cohort	CT	Good
Khafagy et al. [46]	Egypt	3.3	180	1999-2001	60	Retrospective	Cyst.	Fair
Khorana et al. [17]	USA	8.2	360	2004- 2009	2001	Retrospective	CT	Good
Dyer et al. [25]	UK	2.9	360	2009 - 2010	1641	Retrospective	Cyst.	Fair
Ording et al. [47]	Denmark	3.3	360	1995 - 2011	13809	Retrospective	Cyst.	Fair
Wallis et al. [16]	Canada	4.5	720	2002 - 2014	3623	Retrospective	Cyst. + CT	Fair
Walker et al. [24]	UK	1.5	720	1987 - 2012	3152	Retrospective	NA	Fair
Sandhu et al. [48]	USA	1.9	720	1993-95 & 97-99	24861	Retrospective	NA	Fair
Berneking et al. [49]	USA	10.3	------	2000-2010	359	Retrospective	Cyst.	Fair
Mendiola et al. [50]	USA	2.3	----	1995-2003	42	Retrospective	Cyst.	poor
Clement et al. [51]	France	23.2	---	2005-2009	86	Retrospective	Cyst.	Fair
Cardenas-Turanzas et al. [52]	USA	1.4	---	2000-2003	1493	Retrospective	Cyst.	poor

The VTE rate in BC patients varies according to the type of treatment and the use of thromboprophylaxis. Additionally, the mean of VTE rates in BC patients in the UK is lower than the mean of non-UK countries, which indicates that there is a variation in VTE rates in BC patients between the UK and non-UK countries (Figure 4).

According to the author, the VTE rate in BC patients in the UK seems lower than that of non-UK countries, perhaps due to the extended thromboprophylaxis regimens applied post-cystectomy. More data in the UK are required to document the VTE rate in patients who have had a cystectomy and patients treated with cystectomy and chemotherapy. This current review has some limitations, however, as many studies included did not report all variables of interest, e.g., thromboprophylaxis measures and chemotherapy prescribed, but mainly reported VTE rate and the country origin of study. The strengths of this current systematic review include the comprehensive search strategy, the risk of bias assessment and exploring other risk factors for VTE in the BC population.

In summary, this review found the VTE rates in patients with BC who have had cystectomy or chemotherapy are around 2.7% and 5.2%, respectively. There are no data on VTE in BC patients who have had radiotherapy, and very limited data regarding VTE in BC patients who have had cystectomy and chemotherapy in the UK. No data explored the radiotherapy risk factors for the VTE in BC patients.

## Conclusions

This review highlights the fact that the VTE rate in BC varies between studies due to the heterogeneity of risk factors (cystectomy, chemotherapy and thromboprophylaxis) reported. The mean of VTE rates in BC patients who have had cystectomy in the UK seems lower than in non-UK countries. In the UK, there are only two studies that explored the VTE incidence in patients with BC. BC patients have variable regimens and drugs for thromboprophylaxis. VTE rates in patients with BC are affected by the duration of thromboprophylaxis in patients who have had cystectomy. Given the VTE rates in this review, longer-term thromboprophylaxis should be considered postoperatively, so this study supports extended anti-clotting prophylaxis in patients undergoing cystectomy.

Further studies to explore the VTE rate in patients with BC, undergoing cystectomy or receiving multimodality bladder preservation treatment, should be carried out in the UK, to garner more robust data to better protect patients from this debilitating condition.

## Figures and Tables

**Figure 5:**
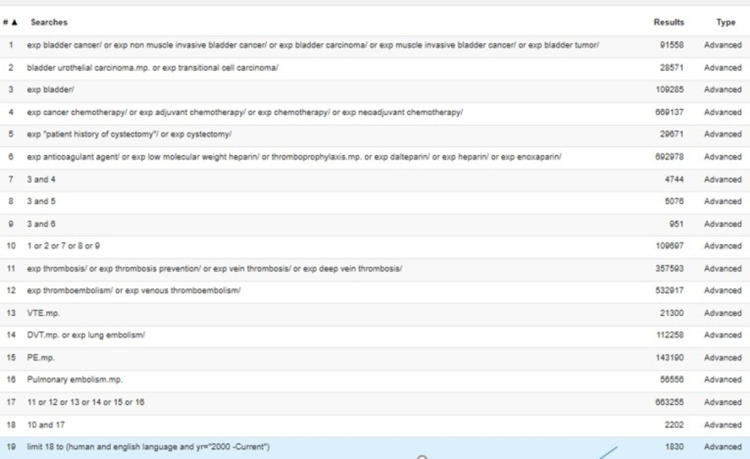
Sample of search of systematic review

## Appendices

Appendix 1: Sample of search of systematic review

Appendix 2: Newcastle-Ottawa quality assessment form for cohort studies

Note: A study can be given a maximum of one star for each numbered item within the Selection and Outcome categories. A maximum of two stars can be given for Comparability.

Selection

1) Representativeness of the exposed cohort

a) Truly representative (one star)

b) Somewhat representative (one star)

c) Selected group

d) No description of the derivation of the cohort

2) Selection of the non-exposed cohort

a) Drawn from the same community as the exposed cohort (one star)

b) Drawn from a different source

c) No description of the derivation of the non-exposed cohort

3) Ascertainment of exposure

a) Secure record (e.g., surgical record) (one star)

b) Structured interview (one star)

c) Written self-report

d) No description

e) Other

4) Demonstration that outcome of interest was not present at the start of the study

a) Yes (one star)

b) No

Comparability

1) Comparability of cohorts on the basis of the design or analysis controlled for confounders

a) The study controls for age, sex and marital status (one star)

b) Study controls for other factors (list) _________________________________ (one star)

c) Cohorts are not comparable on the basis of the design or analysis controlled for confounders

Outcome

1) Assessment of outcome

a) Independent blind assessment (one star)

b) Record linkage (one star)

c) Self-report

d) No description

e) Other

2) Was follow-up long enough for outcomes to occur

a) Yes (one star)

b) No

Indicate the median duration of follow-up and a brief rationale for the assessment above:___________________

3) Adequacy of follow-up of cohorts

a) Complete follow-up - all subjects accounted for (one star)

b) Subjects lost to follow up unlikely to introduce bias- number lost less than or equal to 20% or description of those lost suggested no different from those followed. (one star)

c) Follow up rate less than 80% and no description of those lost

d) No statement

Thresholds for converting the Newcastle-Ottawa scales to AHRQ standards (good, fair, and

poor):

Good quality: 3 or 4 stars in selection domain AND 1 or 2 stars in comparability domain AND 2 or 3 stars in outcome/exposure domain

Fair quality: 2 stars in selection domain AND 1 or 2 stars in comparability domain AND 2 or 3 stars in outcome/exposure domain

Poor quality: 0 or 1 star in selection domain OR 0 stars in comparability domain OR 0 or 1 stars in outcome/exposure domain
